# High toxicity and specificity of the saponin 3-GlcA-28-AraRhaxyl-medicagenate, from *Medicago truncatula* seeds, for *Sitophilus oryzae*

**DOI:** 10.1186/1472-6769-12-3

**Published:** 2012-07-02

**Authors:** Pedro Da Silva, Vanessa Eyraud, Maïté Carre-Pierrat, Catherine Sivignon, Isabelle Rahioui, Corinne Royer, Frédéric Gressent

**Affiliations:** 1 Université de Lyon, INRA, INSA-Lyon, IFR-41, UMR203 BF2I, Biologie Fonctionnelle Insectes et Interactions, 20 ave A. Einstein, Villeurbanne, F-69621, France; 2 Centre de Génétique et de Physiologie Moléculaires et Cellulaires, CNRS UMR 5534, Campus de la Doua, 16 rue Dubois, 69622 Villeurbanne, France et Université Claude Bernard Lyon 1, 43 bd du 11 Novembre, Villeurbanne, 69622, France

**Keywords:** Saponin, Insect, *Medicago truncatula*, *Sitophilus oryzae*

## Abstract

**Background:**

Because of the increasingly concern of consumers and public policy about problems for environment and for public health due to chemical pesticides, the search for molecules more safe is currently of great importance. Particularly, plants are able to fight the pathogens as insects, bacteria or fungi; so that plants could represent a valuable source of new molecules.

**Results:**

It was observed that *Medicago truncatul*a seed flour displayed a strong toxic activity towards the adults of the rice weevil *Sitophilus oryzae* (Coleoptera), a major pest of stored cereals. The molecule responsible for toxicity was purified, by solvent extraction and HPLC, and identified as a saponin, namely 3-GlcA-28-AraRhaxyl-medicagenate. Saponins are detergents, and the CMC of this molecule was found to be 0.65 mg per mL. Neither the worm *Caenorhabditis elegans* nor the bacteria *E. coli* were found to be sensitive to this saponin, but growth of the yeast *Saccharomyces cerevisiae* was inhibited at concentrations higher than 100 μg per mL. The purified molecule is toxic for the adults of the rice weevils at concentrations down to 100 μg per g of food, but this does not apply to the others insects tested, including the coleopteran *Tribolium castaneum* and the Sf9 insect cultured cells.

**Conclusions:**

This specificity for the weevil led us to investigate this saponin potential for pest control and to propose the hypothesis that this saponin has a specific mode of action, rather than acting *via* its non-specific detergent properties.

## Background

Chemical pesticides in general, and insecticides in particular, are increasingly used around the world but are also increasingly stigmatized because of their persistence and their toxicity to non-target organisms (impacting amphibians, aquatic wildlife, beneficial insects, such as bees and ladybirds, and even causing mortality among farmers, particularly in developing countries [1-3]). Crop protection against two very important pests, namely cereal weevils and aphids, is currently carried out almost exclusively by chemical treatments. Some alternative methods exist in the fight against these insects, but they are either much less effective or prohibitively expensive compared with chemical control.

Chemical treatments used to protect stored products are the source of the majority of chemical residues in cereals, subsequently found in processed products. High doses of these residues can be dangerous for consumers. However, the presence of insects or mites is the main cause of refusal, for non-compliance with health regulations, of grain deliveries to the food industry. Hence, it is vital to find new molecules which would have a much less deleterious impact on the environment. One of the most promising sources of such compounds is probably plants, which have developed many ways to fight against insects, as well as against fungal and bacterial attacks, and one of these is the use of insecticidal molecules. These molecules may be found in others organisms, such as in spiders or scorpion venom, but then they are generally toxic to mammals as well. The search for insecticidal compounds in plants, and specifically within those plants that are consumed by mammals, could be a valuable means of developing biopesticides for sustainable and healthy agriculture.

Numerous molecules have yet to be identified in plants in terms of their ability to counter insect, fungal or bacterial attacks. These molecules could be of a proteic nature, including thionins, defensins, lipid transfer proteins, snakins or protease inhibitors. They may also be produced by the secondary metabolism of plants, and many defense molecules are of an alkaloid, saponin or flavonoid type (for review see [4]).

One of the most promising insecticidal molecules is the PA1b peptide, extracted from Legume seeds, which constitutes the main defence molecule against insects in these seeds [5]. PA1b is toxic for some insects, such as cereal weevils, but resistant strains of weevils have been found to exist [6]. However, it is known that the seeds from *Medicago truncatula* contain molecules able to kill both susceptible and PA1b-resistant strains of weevils [7]. By purification of the entomotoxic compound, we found that *M. truncatula* seeds are mainly protected against weevil attack by a specific saponin, rather than by the PA1b peptide, but this toxic effect is currently restricted to the rice weevil among insects.

## Methods

### Biological material and toxicity assays

The extracts used for the toxicity assays were flour of the *M. truncatula* seeds, solvant-extracted flour and purified saponin. All these fractions have been tested on *S. oryzae*, whereas all other organisms were assayed with the purified saponin only.

**Rice weevils (*Sitophilus oryzae*, Coleoptera)** were reared on wheat seeds at 27.5°C and 70% RH. Tests and survival analysis were performed on adults feeding on food pellets (composed of wheat flour and water) incorporating the tested fraction, as described in detail in [7]. LT_50_ values were calculated using the SIMFIT software (http://www.simfit.man.ac.uk). The tests of the juvenile stages of *Sitophilus oryzae* could not be done because the larvae of weevils live inside the wheat grain and could not be grown outside, so we do not have an artificial diet where we can incorporating the toxin.

### The red flour beetle, *Tribolium castaneum* (Coleoptera)

Tests were performed, by mixing the saponin into the standard diet (wheat flour 95%, yeast extract 5%). Then, three groups of 20 adults were deposited on cages, and the mortality was recorded every day.

### The aphid *Acyrthosiphon pisum* (Hemiptera)

Growth and toxicity assays were carried out according to [8]. Briefly, UV-sterilized Parafilm sachets enclosing 500 μL of an artificial diet were made under sterile conditions and placed on a PVC ring. A group of 20 neonate larvae were deposited on day 0 on diet containing or not the tested molecule (three groups per condition). The mortality was then recorded every day.

**The mosquito*Aedes aegypti*** was assayed on two strains: the laboratory strain Bora-Bora, susceptible to all insecticides, and a strain selected from Bora-Bora which is tolerant to Bti Cry toxins (LiTOX strain, [9]). Mosquitoes were reared in standard insectary conditions (27°C, 16 h/8 h light/dark period and 80% relative humidity). Larvae were reared in tap water and fed with standard larval food (hay pellets). Bioassays were performed, in triplicate, in a final volume of 200 μL on 10 calibrated 2nd-instar larvae, with saponin concentrations of 0, 25, 250 and 1000 μg/mL. Mortality was recorded at 24 h and 48 h. Because data were not normally distributed, non-parametric Kruskal-Wallis ranked tests were used to test the strain and dose effects on larval mortality. In addition, Mann–Whitney one-tailed tests were used to compare the mortality, at each dose and each time, with that of the control using R software version 2.5 (R Development Core Team 2005).

***Spodoptera frugiperda*Sf9 cells** were grown at 27°C in Grace’s culture medium, supplemented with 10% foetal calf serum (FCS) and with 10 mg.ml^-1^ gentamicin. Sf9 cells were seeded, in 96-well plates, 24 h prior to the experiments (10 000 cells / well) and were exposed to increasing saponin concentrations for another 24 h or 48 h. Cell viability was determined using the CellTiter-Blue Viability Assay (Promega), according to the manufacturer’s instructions. After addition of the dye, the cells were incubated at 27°C for 4 h. The absorbance, at 570 and 600 nm, was then measured using a microplate reader (MR 7000, Dynatech Laboratories Inc., USA).

***Cænorhabditis elegans*worms**, from the N2 wild type strain, were cultured in liquid growth medium (KH_2_PO_4_ 17.2 mM; Na_2_HPO_4_ 16.8 mM; NaCl 85.6 mM; MgSO_4_ 1 mM, cholesterol 26 μM, with OP50 *E. coli* as the food source) in 96-well plates, under constant agitation. Two adult worms were dispensed in each well using a COPAS BIOSORT robot from Union Biometrica (Massachussets, USA), which allows sorting and dispensing of worms according to their size and optical density. They were grown for 7 days at 15°C. The wells also contained increasing concentrations of the tested molecule. The offspring were observed on each day of the growing period to evaluate the effects of the molecule in terms of growth retardation and toxicity.

***E. coli*DH5α** was grown in LB media at 37°C. For toxicity assays, the saponin was added directly to 1 mL of the media and bacterial growth was monitored by recording the OD at 600 nm for 10 h, starting at t = 0 with an OD = 0.01.

**The yeast*S. cerevisiae****,* strain BY4742, was grown on liquid media (YNB; 20 g.L^-1^ glucose; 0.02 g.L^-1^ His; 0.06 g.L^-1^ Leu; 0.04 g.L^-1^ Lys) at 30°C. For toxicity assays, the saponin was added directly to 1 mL of the media and yeast growth was monitored by recording the OD at 600 nm for 30 h, starting at t = 0 with an OD = 0.01.

### Purification of the saponin

We used seeds from *Medicago truncatula* cv. Jemalong. Seeds were crushed in a Warring blender and sieved through a 0.4 mm mesh to separate the cuticles from the flour. The flour was submitted to successive extractions: first, it was extracted in H_2_O/EtOH (80/20, 10 mL for 1 g of flour) for 2 h, at room temperature and with stirring, and then centrifuged for 10 min at 10 000 × g. The supernatant was dried under vacuum in a Buchi Rotavapor. The resulting powder was resuspended in H_2_O/ACN (40/60, 10 mL for 0.1 g of powder), and immediately centrifuged for 10 min at 10 000 × g. The supernatant was again dried under vacuum.

The powder was resuspended in H_2_O/ACN (40/60), at approximately 15 mg.mL^-1^, and filtered on a 0.45 μm sterile filter. The molecules of the extract were separated by RP-HPLC. The extract was injected into a C18 column (250 × 25 mm, 5 μm, Phenomenex) on an Agilent 1200 HPLC apparatus. The flow was 3.5 mL.min^-1^. The gradient was H_2_O + 0.04%TFA (solvent A) / ACN +0.04% TFA (solvent B) 90/10 for 5 min, then 60% solvent B for 25 min. The elution was monitored using a diode array detector at 210 nm. Each fraction harvested was lyophilized.

The fraction containing entomotoxic activity was resuspended in H_2_O, and then injected in the same column with an elution under isocratic conditions (solvent B 30%). Each fraction harvested was lyophilized and stored dry at −20°C until required.

### Mass spectrometry

All of the mass spectra were obtained using a Thermo LCQ advantage ion –trap spectrometer equipped with an electrospray ionization source. Both positive and negative-ion mass spectra were acquired. Positive-ion ESI was performed using an ion source voltage of −4.0 kV and a capillary offset voltage of 42 V. Nebulization was aided by a coaxial nitrogen sheath gas provided at a pressure of 60 psi and desolvation was aided by the use of a nitrogen counter current gas at a pressure of 12 psi. The capillary temperature was set at 200°C.

Negative-ion ESI was performed using an ion source voltage of 4.0 kV and a capillary offset voltage of −86 V. Again, nebulization was aided by a coaxial nitrogen sheath gas provided at a pressure of 60 psi and desolvation was aided by the use of a nitrogen counter current gas at a pressure of 12 psi. The capillary temperature was set at 200°C.

Mass spectra were recorded over the range 50–2000 m/z. Tandem mass spectra were obtained using automated MS/MS and MS3. MS/MS was performed by isolating the base peak (parent ion) above m/z 1087 and using an isolation width of 2.0, a fragmentation amplitude of 0.6, a threshold set at 15,000 and the ion charge control switched on with the maximum acquired time set at 100 ms. The MS3 was performed by isolating, in the same conditions, the parent ion, initially at m/z 1087 and then we performed the isolation and the fragmentation of the product ion at m/z 911.

### NMR spectroscopy

^1^ and ^13^C NMR spectra were recorded on a 500-MHz Brucker Avance NMR spectrometer equipped with a z axis field gradient unit, using CD_3_OD as the solvent for measurement. Conventional 2D ^1^H-^1^H experiments DQF-COSY (double quantum filtered correlation spectroscopy), HOHAHA (homonuclear Hartman Hahn) NOESY (nuclear overhauser effect spectroscopy) and 2D inverse detected ^1^H-^13^C experiments HSQC (heteronuclear single quantum coherence) [10], HMBC (heteronuclear multiple bond coherence) [11] and HMQC (heteronuclear multiple quantum coherence) [12] were all performed at 293K. The data were processed and analyzed using the Topsin software package.

### Acid hydrolysis of saponin

The saponin (4 mg) was treated with 2 mL of 2 N HCl (methanol-H_2_O, v/v 1:1) under conditions of reflux, at 90°C, for 3 hours. The mixture obtained was extracted with CH_2_Cl_2_ three times to separate the agylcone part. The CH_2_Cl_2_ layer was dried, then the powder was resuspended in H_2_O/ACN (20/80). The aglycone was purified by RP-HPLC. The extract was injected into a C18 column (250 x 4.1 mm, 3 μm, Phenomenex). The flow was 1 mL.min^-1^. The gradient was H_2_O + 0.04%TFA (solvent A) / ACN +0.04% TFA (solvent B) 70/30 to 0/100 for 40 min. 1.25 mg of the aglycone part were retained in order to perform biological assays.

### Protein determination

Protein was measured by the bicinchoninic acid procedure, developed by Pierce, with BSA as a reference.

### Ose determination

1 mL of anthrone solution (200 mg in 100 mL H_2_SO_4_) was added to 0.5 mL of the tested solution on ice. The tubes were covered and vortexed, then the reaction mixture was boiled for 10 min. After cooling the tubes, the OD was read at 585 nm and glucose was used as a reference.

### Sterol visualization

1–2 mg of the dried compound were dissolved in 2 mL of CHCl_3_, followed by the addition of 2 mL of concentrated H_2_SO_4_. After a few minutes, the CHCl_3_ fraction becomes red if sterols are present.

### CMC determination

The CMC (Critical Micelle Concentration) was determined according to [13]. Briefly, 1 μL of 1,6-diphenyl – 1,3,5 – hexatriene (DPH), solubilized in THF, was added to 2 mL of increasing doses of the tested compound in 10 mM MES pH 6. After a 30 min incubation in the dark, fluorescence was determined with an excitation wavelength of 358 nm and an emission wavelength of 430 nm.

## Results

### Purification of the molecule responsible for toxicity

**Figure 1:**
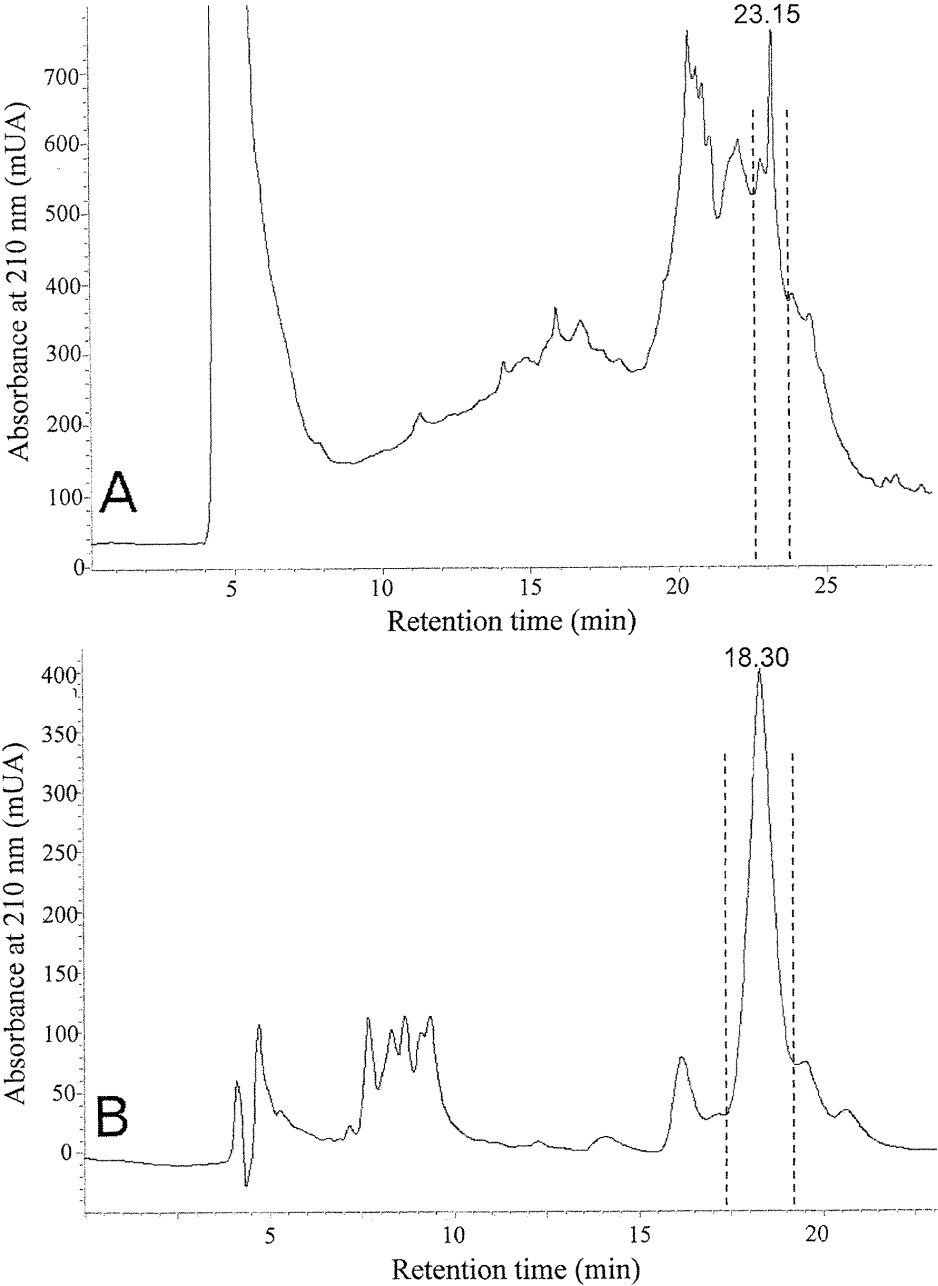
**Chromatograms of*Medicago truncatula*seed extract.A.** Injection into a C18 column (250 × 25 mm, 5 μm, Phenomenex) on an Agilent 1200 HPLC apparatus. The flow was 3.5 mL.min^-1^. The gradient was H_2_O + 0.04%TFA (solvent A) / ACN +0.04% TFA (solvent B) 90/10 for 5 min, then 60% solvent B for 25 min. The elution was monitored using a diode array detector at 210 nm. **B.** The fraction purified in A was injected into the same column with an elution under isocratic conditions (solvent B 30%). Peaks containing entomotoxic activity are indicated.

Flour from the seeds of *M. truncatula* cv. Jemalong displays a strong entomotoxic activity against *S. oryzae* rice weevils (Table 1), displaying a LT_50_ of 7.61 +/− 0.28 days at a dose of 100 mg per g of food. On the basis of this, the toxic compound was purified and the purification steps were followed by biological tests on the rice weevil. Sequential extractions in two different solvents (EtOH 20% followed by an extraction in ACN 60%) causes the resulting fraction to lose 89% of the flour weight while retaining most of the toxic activity (LT_50_ of 3.79 +/− 0.13 days at a dose of 20 mg per g of food for the resulting supernatant, with only slight residual toxicity in the 20% EtOH and 60% ACN pellets). The HPLC chromatogram of this fraction is presented in Figure 1A, and the only toxic fraction was harvested at retention times between 22.6 and 23.8 minutes. Further purification, by isocratic HPLC elution, of this fraction (Figure 1B) resulted in the purification of a single peak at Rt = 18.30 minutes. This isolated peak is the only HPLC fraction displaying an entomotoxic activity, with a LT_50_ of 12.96 +/− 0.52 days at a dose of 200 μg per g of food. The toxic effect was visible at a dose down to 100 μg per g of food (Table 1). The yield of purified compound was approximately 1.25 mg of compound per g of flour (0.12%).

**Table 1:** **Lethal time 50 (LT**_**50**_**) for different fractions of*Medicago truncatula*seed flour on*Sitophilus oryzae***

**Fractions**	**amount per g of food**	**LT**_**50**_**+/− SEM (day)**
*M. truncatula* seed flour	10 mg	> 20
	50 mg	11.03 +/− 0.319
	100 mg	7.61 +/− 0.28
	250 mg	5.33 +/− 0.2
After solvents extraction	20 mg	3.79 +/− 0.13
Purified saponin	20 μg	not toxic
(3-GlcA-28-AraRhaxyl-medicagenate)	100 μg	16.57 +/− 1.89
	200 μg	12.96 +/− 0.52
	400 μg	9.59 +/− 0.39
	800 μg	8.42 +/− 0.25
	1200 μg	6.23 +/− 0.24
	1600 μg	5.63 +/− 0.28

The indicated amount of each powder was added to one gram of wheat flour. The food produced was given to weevils and mortality recorded every day for 20 days.

### Characterization of the toxic molecule

**Figure 2:**
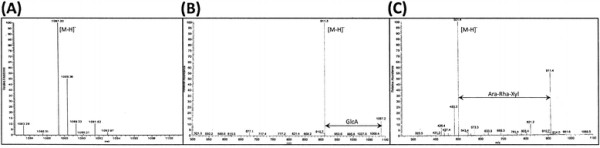
**Negative-ion ESI-mass and tandem mass spectra of 3-O-[β-D-glucuronopyranosyl]-28-O-[β-xylopyranosyl(1 → 4)-α-L-rhamnopyranosyl (1 → 2) - α-L-arabinopyranoside medicagenate (3-GlcA-28-AraRhaxyl-medicagenate).(A)** the ESI-mass spectrum of the isotope distribution of the (M-H)^-^ ion at m/z 1087.33, of 3-GlcA-28-AraRhaxyl-medicagenate, **(B)** MS/MS of 3-GlcA-28-AraRhaxyl-medicagenate, precursor ion is m/z 1087.3, fragment ion at m/z 911.3 correlates to the loss of glucuronic acid (GlcA), **(C)** MS3 of 3-GlcA-28-AraRhaxyl-medicagenate, precursor ion is m/z 911.3, fragment ion at m/z 501.4 correlates to the sequential loss of sugar substituents arabinose, rhamnose and xylose (Ara-Rha-xyl) and thus the fragment ion at m/z 501.4 corresponds to M-H mass of medicagenic acid.

To identify the toxic compound, a mass spectrometry analysis revealed a single (M-H) ^-^ ion peak at m/z 1087.33 (Figure 2A). A protein assay revealed that the molecule is not a peptide; moreover, the toxic activity was not lost after boiling the molecule for 10 minutes. Assays with anthrone and concentrated H_2_SO_4_ were both positive, indicating that the compound was at least partly made up of sugar and sterol. Next we hypothesized that the molecule belonged to the saponin family, and both the ESI/MS/MS analyses (Figure 2B2C) and the ^1^H and ^13^C NMR spectroscopic data of the entomotoxin (Table 2) compound were found to be similar to those reported for a saponin named 3GlcA-28-AraRhaxyl-medicagenate [14]. The entomotoxin compound and the reported saponin [14] have the same ^1^H and ^13^C chemical shits (Table 2). MS/MS and MS3 experiments of the two compounds also produce identical fragment ions at m/z 911.3 and at m/z 501.4 (Figure 2B2C).

**Table 2:** ^**1**^**H et**^**13**^**C NMR data for the saponin 3-GlcA-28-AraRhaxyl-medicagenate**^**a**^**obtained in CD**_**3**_**OD at 298K and 500 MHz**

	^13^C ppm	^1^H ppm
Aglycone (medicagenic acid))		
1	44.4	2.11 *dd* (14.6, 2.3); 1.25^b^
2	70.6	4.29 *m*
3	86.1	4.08 *d* (3.5)
4	53.0	
5	52.8	1.59^b^
6	21.0	1.64^b^, 1.16^b^
7	33.0	1.76^b^, 1.54^b^
8	40.8	
9	49.0	1.60^b^
10	37.1	
11	24.3	2.01^b^, 1.94^b^
12	123.5	5.32 *t* (3.2)
13	144.6	
14	42.8	
15	28.4	1.67^b^, 1.11^b^
16	23.4	2.04^b^, 1.67^b^
17	47.5	
18	42.1	2.93 *dd* (13.5, 4.3)
19	46.8	1.15^b^, 1.12^b^
20	31.4	
21	34.3	1.39^b^, 1.22^b^
22	33.2	1.34^b^, 1.31^b^
23	181.4	
24	13.1	1.40 *s*
25	16.9	1.24 *s*
26	17.4	0.81 *s*
27	25.9	1.16 *s*
28	177.5	
29	33.1	0.91 *s*
30	23.6	0.95 *s*
GlcA		
1	104.9	4.40 *d* (7.7)
2	74.5	3,27^b^
3	77,9	3.30 *t* (9.1)
4	73.0	3.48 *t* (9.1)
5	76.8	3.36 *d* (9.1)
6	173.2	
Ara		
1	93.6	5.69 *d* (3.5)
2	75.3	3.80 t (4.5)
3	70.5	3.90 *m*
4	66.7	3.84 *m*
5	62.9	3.91 *m*, 3.49 *m*
Rha		
1	101.2	5.03 *s*
2	71.8	3.86^b^
3	70.8	3.47^b^
4	83.1	3.55 *m*
5	68.5	3.72 *m*
6	17.3	1.28 *d* (6.2)
Xyl		
1	106.5	4.49 *d* (7.7)
2	75.5	3.19 *dd* (9.1, 5.2)
3	76.8	3.37 *t* ((9.1)
4	70.7	3.56 *m*
5	67.1	3.84^b^, 3.18 *dd* (11.5, 9.4)

^a^ Italic letters indicate the multiplicity of the NMR peaks and J values are in parentheses (Hz).

^b^ overlapping signals.

**Figure 3:**
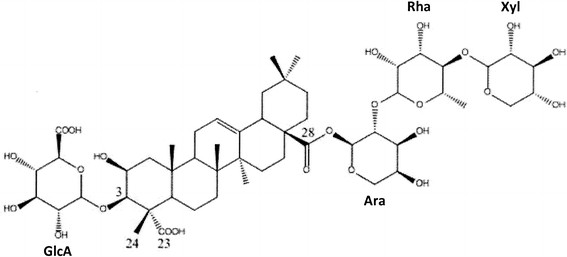
**3-O-[β-D-glucuronopyranosyl]-28-O-[β-xylopyranosyl(1 → 4)-α-L-rhamno-pyranosyl (1 → 2)- α-L-arabinopyranoside medicagenate structure (MW: 1088.5 g/mol).** GlcA, Ara, Rha, Xyl indicate respectively glucuronic acid, arabinose, rhamnose and xylose sugar moieties.

The molecule was, thus, identified as 3-O-[β-D-glucuronopyranosyl]-28-O-[β-xylopyranosyl(1 → 4)-α-L-rhamnopyranosyl(1 → 2)-α-L-arabinopyranoside medicagenate (its molecular formula is C_52_H_80_O_24_ and its CAS registry number is 128192-15-4) (Figure 3).

### CMC determination of the saponin

**Figure 4:**
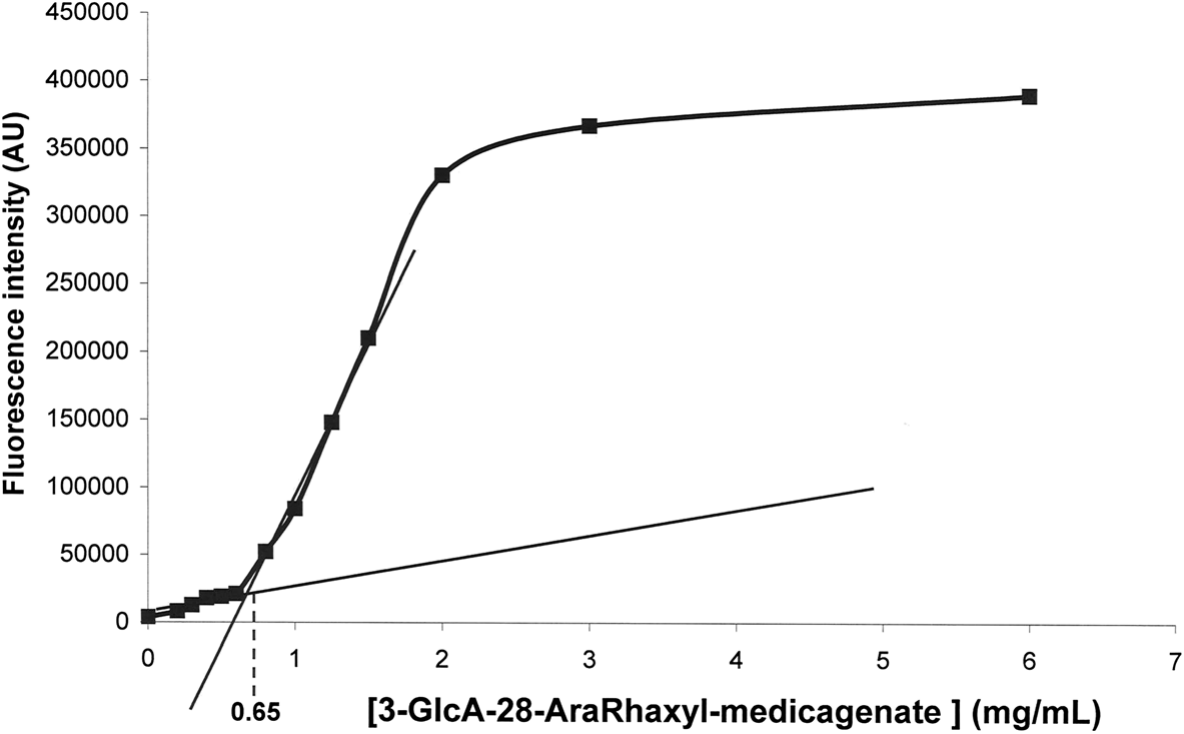
**DPH fluorescence in increasing 3-GlcA-28-AraRhaxyl-medicagenate concentrations.** The calculated CMC was 0.65 mg.ml^-1^ (0.6 mM).

The commercial detergent Chaps was used as a control for the technique. The Chaps CMC was measured as 0.48% (8 mM), identical to that found in the literature [15]. Figure 4 shows DPH fluorescence associated with an increasing amount of the purified saponin, leading to the calculation of a CMC of 0.65 mg.ml^-1^ (0.6 mM).

### Toxicity of the saponin on living organisms

As previously described, the purified saponin is toxic for rice weevils at a concentration as low as 100 μg per g of food (Table 1). Then, the saponin was hydrolysed and the aglycone part of the extraction was confirmed by mass spectrometry. This lipidic part of the molecule was found to have no effect on weevil mortality at a concentration of up to 2 mg per g of food.

A biological test was performed on the red flour beetle, *Tribolium castaneum* (Coleoptera), displaying no mortality on adults. Moreover, the adults lay on the flour containing the purified saponin, and the larvaes develop normally; emergence occurs at the same time as in the saponin-free control diet.

Using the aphid *A. pisum* (Hemiptera)*,* the saponin was tested from 125 to 1000 μg/mL. No mortality was observed during the test, and it was only at the higher dose of 1000 μg/mL that larvae were seen to be smaller and that less honeydew was produced.

Larvae of two strains of the mosquito *Ae. aegypti* (Diptera) were assayed at 0, 25, 250 and 1000 μg.mL^-1^ of saponin. There was no effect associated with the mosquito strain or the saponin concentration on mortality: even at the highest dose, mortality was no different from the control.

We next tested the saponin on an insect cultured cell system, the Sf9 cells from *Spodoptera frugiperda* (Lepidoptera). We used the *Pisum sativum* PA1b toxin, at 5 μg.mL^-1^ , as a positive control which led to 100% cell death 24 h after addition without any disruption of the cell membrane. Using doses of the *M. truncatula* saponin up to 1000 μg.mL^-1^, we have demonstrated that cultured cells were not affected by this toxin as no cell death was observed 24 or 48 h after addition of the saponin. On the other hand, an experiment using the commercially available saponin from *Quillara saponaria* (SIGMA Ref. S4521) showed a strong toxic effect, with a DL_50_ of 1.25 μg.mL^-1^ and total disruption of the cell membrane (data not shown).

We tested the susceptibility of *Cænorhabditis elegans* worms to the saponin from *M. truncatula*. Three doses were tested: 0.02, 0.2 and 2 mg per g of media. For the three doses, no embryonic, larval or adult mortality was observed. There were no effects on adults, and it was only at the highest dose that we observed a delay in larval development of up to one and a half developmental stages (data not shown).

**Figure 5:**
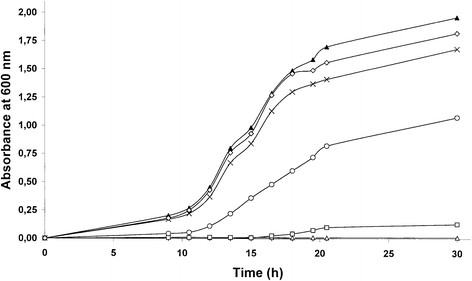
**Effect of 3-GlcA-28-AraRhaxyl-medicagenate on the growth of the yeast*Saccharomyces cerevisiae.***Yeast growth was followed by the absorbance, at 600 nm, of 1 mL of yeast culture in YNB media. An increasing concentration of saponin from *M. truncatula* was added to the media; 0 mg.mL^-1^ (▲); 0.025 mg.mL^-1^ (⋅); 0.05 mg.mL^-1^ (x); 0.1 mg.mL^-1^ (⋅); 0.25 mg.mL^-1^ (❏); 0.5 mg.mL^-1^ (;△); 1.5 mg.mL^-1^ (+).

The growth rate of *E. coli* bacteria was measured in the presence of the saponin. For doses up to 2 mg.mL^-1^, no differences were observed in bacterial growth in the presence or absence of the saponin. By contrast, the saponin strongly affected the growth of the yeast *Saccharomyces cerevisiae*. The results presented in Figure 5 clearly show an inhibition of yeast growth, compared to the control, for doses higher than 0.1 mg.mL^-1^, with total growth inhibition at 0.25 mg.mL^-1^ and higher concentrations.

## Discussion

With increasing concern about the effects of chemical pesticides on the environment and on human health, the search for molecules with pesticidal activity but without, or at least with only minor, adverse effects becomes more and more important. A number of molecules of plant origin with antimicrobial or antifungal activity are currently available, but only a few molecules have insecticidal properties, especially against insects which damage stored products, such as cereal weevils.

The observation that *M. truncatula* seed flour had a rapid lethal effect on the rice weevil, and that this mortality was not due to PA1b because the PA1b resistant strain was killed as well [7], led us to investigate this Legume seed further. We succeeded in isolating the entomotoxic compound, which was found to belong to the saponin family and was identified as 3-GlcA-28-AraRhaxyl-medicagenate. This compound has already been described in the literature [14], but without any demonstration of its toxic properties, although its potential use against pea aphids has been previously suggested [16]. The use of saponins as natural insecticides is an idea that is gaining importance [17,18]. As with other saponins, 3-GlcA-28-AraRhaxyl-medicagenate displays detergent properties and has a CMC of about 0.6 mM. This property could explain the toxicity of the *M. truncatula* saponin on yeast, even though toxicity occurs at concentrations below the CMC. Indeed, many saponins display antifungal activities [19] and, to date, the mechanism of action of these compounds on fungi has been found to be due to the detergent function, via an interaction with a sterol in the membrane [20]. Although nematocidal activity of saponins from *Medicago* spp. has been observed at relatively low doses (0.5 mg.mL^-1^ of a mixture of saponin isoforms) on *Xiphinema*[21], the purified saponin of *M. truncatula* is not toxic for the nematode *C. elegans*.

However, the most promising and interesting property of the *M. truncatula* saponin remains its activity against the rice weevil *S. oryzae,* even at relatively low concentrations. Some examples of the insecticidal activities of members of the saponin family have already been described: the anti-feeding activity of a pea saponin on weevils [22]; the action of triterpenoid saponin, from *Barbarea vulgaris,* on *Plutella xylostella*[23]; and the non-specific toxic effects of different saponins on *Spodoptera littoralis* and *Acyrthosiphon pisum*[24]. Other saponins have been described as being lethal for rice weevils, but at higher doses [25,26].

The most surprising result is that *S. oryzae* is the only tested insect found to be sensitive to the saponin. Even *T. castaneum*, another coleopteran, and Sf9 cultured cells were fully non-susceptible to a high saponin concentration. This last result demonstrated that 3-GlcA-28-AraRhaxyl-medicagenate is not cytotoxic for Sf9. However, several saponins display cytotoxic properties against cancer cells and are considered as potential anti-cancer agents [27,28]. The saponins from *Q. saponaria*, which rapidly killed Sf9 cells, also displayed cytotoxic activity [29].

The saponin under investigation here has a specific toxicity on rice weevils and not on other insects. Interestingly, *Aedes aegyptii* is susceptible to *Q. saponaria* saponin at a dose of 0.8 mg.mL^-1^[30], and to other saponins [31], but not to the *M. truncatula* saponin. Furthermore, in *M. truncatula* seeds, a number of other saponins exist [14,32] and, out of all the HPLC fractions obtained, only one fraction, and even only one saponin molecule, displayed toxicity on weevils. This high specificity of 3-GlcA-28-AraRhaxyl-medicagenate for the rice weevil suggests that the mechanism of action could not be explained simply by a detergent action, and that a more specific mechanism, putatively involving the existence of a specific receptor on the insect, may exist. Although the antifungal activity of saponins seems to be due to an interaction with membrane sterols, a number of mechanisms of action for the other biological activities of saponins have been suggested.

The next step in this investigation will be to determine the precise mode of action of the saponin on the rice weevil. This could open the way to the discovery of a new target for bioinsecticides. The high specificity of 3-GlcA-28-AraRhaxyl-medicagenate for rice weevils, together with a relatively low lethal concentration and the absence of any effects on other organisms, such as bacteria or nematodes, are interesting properties in the goal to combat weevils during cereal storage using low doses.

## Conclusions

The flour from *Medicago truncatula* seeds was found to be highly toxic for the rice weevil *Sitophilus oryzae*. The insecticidal compound was purified and identified as a saponin, 3-GlcA-28-AraRhaxyl-medicagenate. The molecule displayed an antifungal activity, but no bacterial or nematocidal toxicity. However, the most important result is that 3-GlcA-28-AraRhaxyl-medicagenate induced mortality in the rice weevil, but not in the other tested insects, at doses down to 0.1 mg.ml^-1^, suggesting a mechanism of action involving a specific receptor present in weevils.

## Abbreviations

PA1b, Pea Albumin 1 subunit b; CMC, Critical Micelle Concentration.

## Authors’ contributions

PDS carried out the identification of the saponin *via* mass spectrometry and ^1^H and ^13^C NMR spectroscopic analyses. VE performed and analysed the toxic assays on the yeast *S. cerevisiae* and the bacteria *E. coli*. MCP carried out the bioassays on *Cænorhabditis elegans* worms. CS participated in the purification of the saponin. IR performed the purification of saponin, biochemical tests and bioassays on *Spodoptera frugiperda* Sf9 cells. CR participated and analysed the bioassays on *Cænorhabditis elegans* worms. FG conceived of the study and participated in its design and coordination and drafted the manuscript. All authors read and approved the final manuscript.
